# Structural and Functional Investigation and Pharmacological Mechanism of Trichosanthin, a Type 1 Ribosome-Inactivating Protein

**DOI:** 10.3390/toxins10080335

**Published:** 2018-08-20

**Authors:** Wei-Wei Shi, Kam-Bo Wong, Pang-Chui Shaw

**Affiliations:** Centre for Protein Science and Crystallography, School of Life Sciences, The Chinese University of Hong Kong, Sha Tin New Town, Hong Kong, China; Shiww@cuhk.edu.hk (W.-W.S.); kbwong@cuhk.edu.hk (K.-B.W.)

**Keywords:** TCS, ribosome-inactivating protein, ribosomal stalk P protein, multiple pharmacological activities, mechanism

## Abstract

Trichosanthin (TCS) is an RNA *N*-glycosidase that depurinates adenine-4324 in the conserved α-sarcin/ricin loop (α-SRL) of rat 28 S ribosomal RNA (rRNA). TCS has only one chain, and is classified as type 1 ribosome-inactivating protein (RIP). Our structural studies revealed that TCS consists of two domains, with five conserved catalytic residues Tyr70, Tyr111, Glu160, Arg163 and Phe192 at the active cleft formed between them. We also found that the structural requirements of TCS to interact with the ribosomal stalk protein P2 C-terminal tail. The structural analyses suggest TCS attacks ribosomes by first binding to the C-terminal domain of ribosomal P protein. TCS exhibits a broad spectrum of biological and pharmacological activities including anti-tumor, anti-virus, and immune regulatory activities. This review summarizes an updated knowledge in the structural and functional studies and the mechanism of its multiple pharmacological effects.

## 1. Introduction

Trichosanthin (TCS) is extracted from the root tuber of the Chinese medicinal herb *Trichosanthes kirilowii* Maximowicz (Tian Hua Fen), which has been used clinically as an abortifacient drug in early and mid-gestation for centuries. Apart from inducing midterm abortion [1], TCS displays other encouraging potentials for future clinical applications for its anti- human immunodeficiency virus (HIV) [2,3] and anti-tumor [4,5] activities. Because of these findings, TCS has received increased attention, and its structure and function and pharmacological properties have been further investigated [6,7,8,9,10].

The precursor of TCS is a 27 kDa protein consisting of 289 aa. The active form of TCS is obtained after the N-terminal 23 aa signal peptide and C-terminal 19 aa peptide are removed [1,11]. TCS belongs to type 1 ribosome-inactivating protein (RIP), which is a single chain polypeptide that can inactivate eukaryotic ribosomes by cleaving the *N*-glycosidic bond at adenine-4324 of 28 S rRNA [12]. This halts the protein synthesis function of the ribosome and ultimately results in the cell death [13,14].

TCS manifests attractive pharmacological properties for its anti-tumor [9,15,16,17], anti-virus [2,18] and immunoregulatory activities [19,20]. Recently, it has been found that TCS not only exhibits a very high in vitro antitumor activity to common tumor cells, it can also kill multidrug-resistant cancer cells [21]. Besides having anti-HIV effect [22], TCS also exhibits a promising inhibitory effect on Herpes simplex virus (HSV) [23] and Hepatitis B virus (HBV) [24]. Moreover, TCS can induce immunosuppression on the non-toxic T-lymphoproliferative responses in humans [25,26], up-regulate interleukin (IL)-4 gene expression and suppress interferon (IFN)-γ gene expression. TCS also regulates the expansion of CD4^+^CD25^+^ regulatory T cells [27,28] and the researchers found that TCS can prevent allograft rejection and prolong graft survival duration in a murine skin transplantation model [20,28]. In this review, we summarize the recent progress on the structure–function study of TCS, its various pharmacological properties and potential therapeutic applications.

## 2. The Structural Investigation of TCS

### 2.1. The Structural Feature and Ribosomal Interaction Mode of TCS

**Figure 1:**
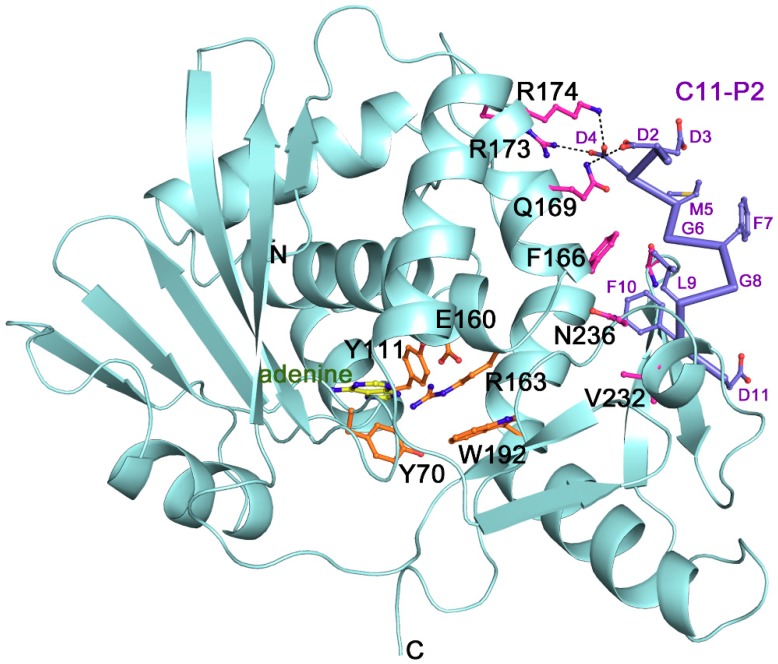
The overall structure, active site and ribosomal P protein binding site of trichosanthin (TCS) (PDB code: 2JDL and 1GIS). The conserved active site residues are shown in orange sticks. Adenine is shown in yellow sticks. The P2 binding residues are shown in pink sticks. The C11-P2 peptide is shown as purple sticks. Hydrogen bonds are highlighted with black dash lines.

The crystal structure shows that TCS consists of two domains [29]. The large N-terminal domain contains six alpha-helices, a six-stranded sheet, and an antiparallel beta-sheet. The small C-terminal domain has the largest distinct bent alpha-helix. Five structurally conserved catalytic residues Tyr70, Tyr111, Glu160, Arg163 and Phe192 (Figure 1) are located on the active cleft between these two domains. The orientation of tyrosine ring of the Tyr70 is flexible and this forms a π-π stacking interaction with the adenine ring of the substrate to facilitate ligand binding [30]. Phe192 lies at the bottom of the active site pocket, the indole ring could stabilize adenine binding [31].

TCS was identified to bind the ribosomal stalk P proteins and L10a [32,33]. In eukaryotic ribosomes, a pentameric complex of ribosomal P proteins, with one P0 and two P1 and P2, forms a flexible stalk to recruit the elongation factors to facilitate the protein synthesis [34,35,36]. All P proteins possess a highly conserved amino acid sequence rich in acidic residues in their C-termini [33], and this sequence is found to be involved in the stalk activity [37] and interacts with several RIPs [38,39,40,41]. Our structural study revealed the binding mode of TCS toward the C-terminal peptide (C11-P) of eukaryotic ribosomal P protein [42]. The ribosomal protein binding site is located between the anti-parallel beta-sheets 9 and 10. Three basic residues Lys173, Arg174 and Lys177 in TCS form charge–charge interactions with the acidic DDD motif while a hydrophobic pocket lined by Phe166, Leu188 and Leu215 accommodates the LF motif of C11-P2 (Figure 1).

The NMR structure study of the stalk protein complex of P1/P2 heterodimer and biochemical analyses demonstrated that the flexible C-terminal tails of eukaryotic ribosome stalk can form an arm-like structure that extend with a radius up to ~125 Å [43,44,45]. The long flexible linker presumably plays an important role in reaching out to capture the elongation factors nearby [46,47]. Truncation of the linker region results in great reductions of depurination activity [48]. It has also been shown that the C-terminal tails and flexible linker of the ribosomal stalk are essential for binding eukaryotic factors 2 (eEF2) [46,47,49,50]. In archaea, the C-terminal tail of archaeal P protein aP1 has been identified to bind the initiation factor aIF5B as well as elongation factors aEF-1α and aEF-2 [37]. This interaction is further identified to be mediated by the conserved hydrophobic amino acids of the C-terminal tail of archaeal stalk proteins [37]. The crystal structure of a complex of C-terminal tail of aP-aEF1α·GDP revealed the same conclusion; that the C-terminal tail of aP1 interacts with domains 1 and 3 of aEF1α mainly mediated by hydrophobic interactions [51]. Phylogenetic and functional analyses suggested that the eukaryotic stalk P protein may directly interact with eEF1α, in a manner like the binding between archaeal aP1 and aEF1α [51].

Taken together, the structural and biochemical observations suggest that RIPs and eEF2 may compete for binding to the flexible C-terminal tail of ribosomal stalk P protein. Therefore, we proposed that eukaryote-specific RIPs may hijack the elongation-factor recruiting function of ribosomal stalk to access to the α-SRL [52].

### 2.2. The Possible Pell-Entry Pathway of TCS

**Figure 2:**
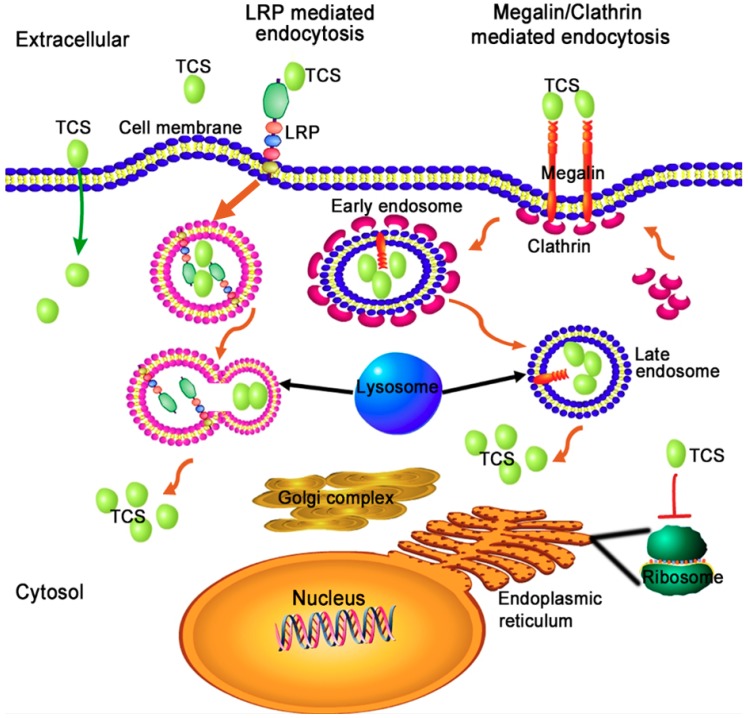
The proposed cell-entry and intracellular trafficking pathway of TCS. Through interacting with phospholipids of cell membrane, lipoprotein receptor-related protein (LRP) receptor and Megalin/Clathrin surface architectures, TCS is transported into the cytosol.

It is difficult for TCS to enter cells because the protein lacks a lectin-binding domain found in ricin. It is found that both receptor-mediated endocytosis and non-specific entry may be involved in the cell entry route of TCS [53] (Figure 2). TCS could interact with low-density lipoprotein receptor-related protein (LRP), megalin and through clathrin-coated vesicles to enter into mammalian cells [54,55]. The last seven amino acids at the C-terminal of TCS could interact with the phospholipid bilayer via electrostatic interaction under acidic conditions [56,57]. This interaction leads to membrane fusion, thus facilitating the translocation of TCS into the cytosol [56,57]. It was found that deletion of the TCS C-terminus almost completely abolishes the destabilizing effect of TCS on the phospholipid bilayer and significantly reduces both its RIP activity in vitro and cytotoxicity in vivo [57]. It has been assumed that TCS interacts with these carriers/receptors to form endosomes, the enveloped TCS is released into the cytosol by lysosome digestion and gradually transported to ribosomes to perform its RIP activity. Internalized TCS could cause vesicle leakage, membrane fusion [56] of subcellular organelles, and can induce the autophagy and apoptosis of cancer cells [58,59,60,61]. Recent findings suggest that the intracellular traffic of TCS into mammalian cells is a key step for its biological activities, because the cytotoxicity of TCS is dependent on its intracellular concentration [62]. TCS competitively binds to a surface clathrin adaptor GGA (Golgi-localized, gamma-adaptin ear homology, ARF-binding proteins) of Golgi to affect the subsequent signal transduction, thus enhancing apoptosis in HepG2 hepatoma cells [63].

## 3. TCS Possesses Multiple Pharmacological Properties

TCS has a broad spectrum of biological and pharmacological activities, including induction of abortion, inhibition of tumor cell proliferation via render protein synthesis, anti-viral activity, effective against a variety of tumors and immunomodulatory activity. These have been reviewed previously [53]. Here, we summarize the recent progresses on these pharmacological activities and the important signal pathways for TCS to exert its pharmacological effects.

### 3.1. Anti-Viral Activity of TCS

#### 3.1.1. Anti-HIV-1 Activity

Previous studies reported that TCS selectively inhibits the replication of HIV virus type 1 (HIV-1) in both acutely infected T-lymphoblastoid cells and chronically infected macrophages in vitro [64]. Phase I/II clinical trials with TCS alone or in combination with other anti-HIV drugs, zidovudine or dideoxinosine, showed that TCS could decrease serum HIV-1 p24 antigen level and increase the percentage of CD4^+^ cells in patients with acquired immunodeficiency syndrome (AIDS) and AIDS-related complex [65,66]. It is generally assumed that the antiviral activity of TCS is related to its RIP activity. It was found that TCS variants without catalytic residues and residues close to the active site could lose almost all of their anti-HIV-1 activity [22]. Intriguingly, the C-terminal addition variants of TCS, within the 19 amino acid extension and an ER retrieval signal sequence KDEL at the C-terminus of TCS, retained all RIP activity but lost most of the anti-HIV-1 activity, while the TCS C-terminal deletion variants lost all activities [67]. Combined with our structural findings, TCS C-terminal deletion variants may damage the ribosomal stalk P2 binding interface thus decrease its RIP activity and anti-HIV-1 activity. On the other hand, addition of amino acids at the C-terminal does not destroy the P2-binding pocket, hence there was no effect on its RIP activity while the anti-HIV-1 activity was blocked. These results suggested that RIP activity of TCS may have significant correlation with its anti-HIV-1 property. Another observation demonstrated that TCS can penetrate into HIV-1 viral particles, and several residues, FYY140-142, D176, and K177 were identified as key amino acid residues for mediating membrane translocation process into HIV-1 virions [68]. The penetration of TCS exerts no obvious effect on viral integrity. However, TCS-enriched HIV-1 virions were severely impaired in its viral infectivity [69]. It was then found that TCS transiently binds and depurinates the long terminal repeats of HIV-1, which may be responsible for the inhibitory activity on HIV-1 integration [70].

#### 3.1.2. Anti-Hepatitis B Virus Activity 

The plant *Trichosanthes kirilowii* has a long history of clinical use for treating hepatitis B virus (HBV) in China. As the main component in the aqueous extract of Trichosanthis Radix, TCS is found responsible for the anti-HBV effect [24]. TCS has significant and effective dose-dependent and time-dependent inhibition of the expression of HBsAg and HBeAg antigen in HepG2.2.15 cells [24]. However, the molecular mechanism of this inhibitory action to HBV is still elusive.

#### 3.1.3. TCS has Protective Effect against Herpes Simplex Virus in Animal Model

**Figure 3:**
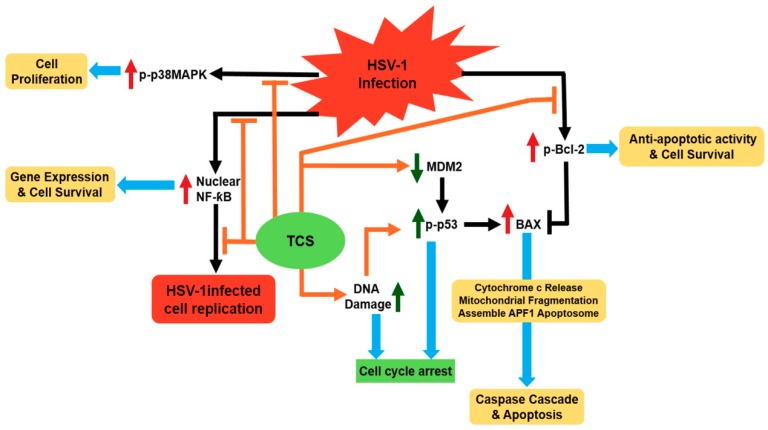
Proposed anti-HSV (Herpes simplex virus) mechanism of trichosanthin via the modulation of key signaling pathways. Black and orange arrows represent the activation of signal transduction receptors, blunt arrows represent inhibition of signal transduction receptors initiated by HSV infection (black) and TCS treatment (orange). Red/green upward arrows and downward arrows represent the upregulation and downregulation effect triggered by HSV infection (red) and TCS treatment (green). Blue arrows indicate the outcomes of signal transduction.

Herpes simplex virus (HSV) is responsible for a broad range of human diseases [71]. Type 2 HSV infection is frequently found associated with HIV-1 infection and can be lethal to AIDS patients [72]. All the clinically available anti-viral drugs such as interferons (INFs), acyclovir (ACV), vidarabine, ganciclovir and foscarnet have adverse complications and could induce resistance [71]. A previous study has found that TCS can synergistically enhance the anti-HSV effect of INFα2a and ACV at cellular level [73]. TCS has a protective effect against HSV-1 induced infection in mice model [23]. It has been further shown that TCS could suppress the HSV-1 viral replication both in Vero cells and human epithelial carcinoma Hep-2 cells [74,75]. TCS may exert its anti-HSV virus activity via modulating three important signal pathways, namely p38 MAPK/Bcl2, nuclear factor-kappaB and p53 pathways (Figure 3) [74,76]. TCS can suppress the elevation of p38 MAPK and Bcl-2 induced by HSV-1 infection to reduce viral replication in Vero cells [74]. It also suppresses the activation of NF-kappaB and regulation of p53-dependent cell death in infected Hep-2 cells [76].

### 3.2. The Anti-Tumor Activities of TCS

#### 3.2.1. TCS Inhibits Various Tumor Cells

Previous studies have indicated that TCS exerts a selective and high toxicity for a broad range of tumor cells [53,77] in vitro and in vivo (Table 1). Recent studies have demonstrated TCS can effectively inhibit the proliferation and viability of cancer cells by inducing apoptosis in several tumor cell lines and in animal models.

#### 3.2.2. The Possible Anti-Tumor Mechanism of TCS

**Figure 4:**
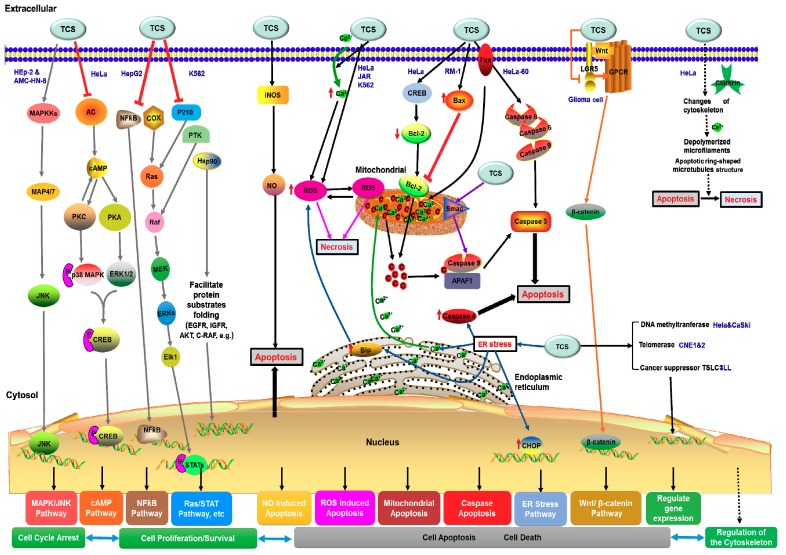
Plausible mechanism of anti-tumor and TCS-induced apoptosis pathways. Arrows represent the activation of signal transduction receptors, red blunt arrows represent inhibition of signal transduction receptors. Small upward/downward red arrows represent the upregulation/downregulation initiated by TCS treatment. Ca^2+^ is calcium ions; c represents the cytochrome c; P represents phosphorylation.

TCS is known to induce apoptosis of various tumor cell lines. Previous reviews have summarized that TCS could regulate various signaling pathways to determinate the tumor cell fates and induce apoptosis of tumor cells [10,91], including nitric oxide (NO)-mediated apoptosis pathway [92], oxidative stress [58,59], cAMP signaling pathway [93,94] and mitochondrial and endoplasmic reticulum stress signaling pathways [83]. Other subcellular organelles such as ribosomes, microfilaments and microtubules, also play respective and cooperative roles in the TCS-induced apoptosis pathway [10]. TCS can also alter the expression of apoptosis-related genes, regulate cytoskeleton structure, reduce the proliferation or viability of tumor cells, and activate the extrinsic and intrinsic apoptotic pathways [61,83]. Recent studies demonstrated that TCS can inhibit tumor cell proliferation, invasion and migration through modulating the Wnt/beta-catenin signaling pathway [90], or causing a reduction in telomerase activity, restoring the expression of methylation-silenced tumor suppressor genes and promoting Smac demethylation [87,95,96] to regulate key components of other signal pathways to inhibit the tumor cell growth, induce cell apoptosis and autophagy. Besides inhibiting tumor cells directly, TCS also enhances anti-tumor immunity via regulating the expression of tumor suppressors in cancer cells and modulating its interaction with protein interacting partners in T cells [86]. Combining all these reports, TCS generally exploits apoptosis and autophagy related pathways to exert its anti-tumor activity. We may categorize the anti-tumor mechanism into two types; apoptosis related and autophagy related strategies. According to the antitumor effects of TCS, the apoptosis related type can be further classified into four types (Figure 4).

##### Apoptosis Related Anti-Tumor Mechanism

(1) Inhibition of Tumor Cell Proliferation

TCS may activate the JNK/MAPK signaling pathway to inhibit the cell viability and proliferation of human epithelial type 2 (HEp-2) and AMC-HN-8 human laryngeal epidermoid carcinoma cells [97]. In HeLa cells, TCS can suppress adenylyl cyclase (AC) activity and initiate the increase of cytosolic calcium to reduce cyclic AMP (cAMP) levels [94]. The decreased cAMP level could inhibit the PKA and PKC activities, suppress the cAMP/PKA and PKC/MAPK signaling pathways to attenuate the phosphorylation of the downstream transcriptional factor cAMP response element-binding (CREB) protein [93,98]. Furthermore, TCS down-regulates nuclear factor kappa B (NF-kB) and cyclooxygenase-2 (COX-2) expression in hepatoma HepG2 cells [99,100]. This induces rapid decline of p210 (Bcr-Abl), protein tyrosine kinase (PTK), and heat shock protein 90 (Hsp90) in chronic myelogenous leukemia K562 cells [101]. TCS eventually regulates all these downstream proliferation-associated genes and proteins to inhibit the proliferation of cancer cells (Figure 4).

(2) Induction of Intrinsic and Extrinsic Apoptosis in Tumor Cells

TCS treatment could induce elevation of intracellular calcium ions (Ca^2+^) and ROS production in HeLa [59,102], JAR [94] and K562 cell lines [83]. The production of ROS promotes the activation of executioner caspase 3 via mitochondrial apoptosis pathway [58,59]. In TCS-treated HeLa cells, CREB was activated and Bcl-2 protein content was decreased. On the other hand, TCS treatment upregulated Bax expression also leading to the inhibition of Bcl-2 in murine prostatic cancer RM-1 cell [102]. The inhibitory effect on Bcl-2 would trigger cytochrome c release from mitochondria, thus inducing the apoptosis pathway. Besides, TCS can activate the caspases 6, 8 and 9 and lead to Smac release from mitochondria into the cytosol to induce apoptosis in HeLa-60 cells through a Fas-mediated pathway [83]. TCS elevated the nitric oxide synthase (iNOS) expression level and induced the augmentation of nitric oxide (NO) and activated the NO-mediated apoptosis pathway to inhibit antigen-specific T cell expansion [92]. Moreover, TCS upregulated the chaperone BiP and transcription factor CHOP, and also activated caspase 4 in HeLa-60 cells thus triggering the endoplasmic reticulum (ER) stress apoptotic pathway [83]. In addition, TCS treatment can markedly decrease the expression level of leucine rich repeat containing G-protein-coupled receptor 5 (LGR5) and repress key proteins in the Wnt/beta-catenin signaling pathway to induce apoptosis in glioma cells (Figure 4), thereby inhibiting glioma cell proliferation, invasion and migration [90].

(3) Regulation of Apoptosis-Associated Genes

Recent reports showed that TCS could inhibit DNA methyltransferase 1 (DNMT1) and restore the expression of methylation-silenced tumor suppressor genes in adenomatous polyposis coli (APC) and tumor suppressor gene in lung cancer 1 (TSLC1) [95]. TCS acts as a demethylation agent promoting mitochondrial protein Smac demethylation and increases its expression in cervical cancer cells [96]. In nude mice, TCS suppresses telomerase activity and induces cell apoptosis to inhibit the growth of nasopharyngeal carcinoma cell lines, CNE1 and CNE2 [87]. TCS not only affects tumor cells directly, but also enhances anti-tumor immunity via regulating the expression and modulating the interaction of tumor suppressor TSLC1 and T cell-associated molecule CRTAM in the 3LL Lewis lung carcinoma tumor model [86] (Figure 4).

(4) Regulation of Cytoskeleton

TCS can induce specific changes, such as depolymerizing microfilaments (MF) and ring-shaped microtubules (MT) structure in cytoskeleton configuration in apoptotic HeLa cells [103] (Figure 4). MF rearrangement could decrease actin and tubulin gene expression levels and lead to execution of HeLa cell apoptosis and the shift from apoptosis to necrosis [103].

##### Autophagy-Related Anti-Tumor Mechanism 

Recent studies have demonstrated that TCS exerts significant anti-tumor effect on human gastric cancer MKN-45 cells via up-regulation of the autophagy protein 5 (Atg5), and conversion of the autophagosome marker LC3 I to LC3 II, then activates NF-kB/p53 pathway, thereby inducing the generation of reactive oxygen species (ROS) to induce gastric cancer cell autophagy [104]. However, it is unclear if the TCS-induced apoptosis or autophagy is dependent on its *N*-glycosidase activity [105,106].

### 3.3. The Immunomodulatory Activity of TCS 

A previous study showed that TCS is a potent immunosuppressive protein which could affect humoral immunity through regulating the ratio of immunoregulatory T lymphocytes cells [19,107] and induce immunosuppression of the non-toxic T-lymphoproliferative responses [25,26]. It has been shown that TCS could inhibit the immune response and specifically induce the expression of cytokines of T helper 2 immune response pathway in mouse splenocytes [108], effectively preventing allograft rejection and prolonging graft survival in a murine skin transplantation model [20]. TCS could up-regulate interleukin (IL)-4 gene expression while suppressing interferon (IFN)-γ gene expression in TCS-immunized mice [27]. It was further shown that TCS plays an important role for the expansion of CD4^+^CD25^+^ regulatory T cells, thus prolonging survival duration and preventing graft-versus-host disease in the mice model [28]. It has been found that TCS could affect the immune effector, class I-restricted T cell-associated molecule (CRTAM) in effector T cells in lung cancer model [86]. These results also suggest the potential therapeutic value of TCS for transplantation rejection and other inflammatory diseases.

## 4. TCS-Derivatives Are Promising Therapeutic Agents

To promote the potential use of TCS as a therapeutic agent, there are attempts to use different approaches to modify TCS as summarized in a previous review [16]. Monoclonal antibody-conjugated TCS changes its specificity and could enhance its antitumor efficacy [10]. TCS-conjugated to anti-hepatoma monoclonal antibody has specific cytotoxicity and effective anti-tumor activity to human hepatoma cells [109]. It has been found that the immunogenicity of TCS is reduced and the biological activity has not been altered by modifying the epitopes of TCS [53]. So far, antigenicity and other side effects, such as poor tumor targeting, short half-life, insufficient tumor accumulation and cell penetration, have precluded further clinical translation of TCS [110]. Efforts to reduce the antigenicity of TCS by molecular manipulation and coupling to PEG have been made [66]. Site-directed PEGylation of TCS retains its anti-HIV activity with reduced potency in vitro [66]. The further bioengineered PEGylated matrix metalloproteinase (MMP)-switchable cell-penetrating TCS showed potent prodrug-like feature and effective synergy effect with paclitaxel in treating multidrug resistance cancer both in vitro and in vivo [21]. Another study utilized a nanotechnology-based co-delivery of TCS protein and albendazole [63] as a combination therapy to overcome drug resistance and inhibit tumor metastasis. Besides, a 15-aa-long TCS-derived peptide can suppress type 1 immune responses, through TLR2-dependent activation of CD8 (+) CD28 (−) Tregs, as effectively as full-length TCS without exhibiting cytotoxicity [111]. Researchers also attempted to explore the potential application of TCS in cancer immunotherapy. They found that TCS may sensitize HepG2 tumor cells to cytotoxic T lymphocyte-mediated tumor cell apoptosis and enhance the efficacy of cancer immunotherapy in a nude mice model [112]. A recent study also showed that TCS fused with intracellular delivery vehicles can drastically improve the TCS intracellular efficiency, specificity and enhance its cytotoxicity. For example, TCS fused with a heparin-binding peptide (HBP) altered its intracellular delivery route and increased its cytotoxicity to tumor cells [113]. After combination with a human derived cell-penetrating peptide HBD, TCS showed an efficient delivery into tumor cells [114].

## 5. Conclusions

TCS possesses a number of biological activities, including anti-virus, anti-tumor and immune-regulatory functions. Existing reports showed that the anti-tumor properties and the mechanism of TCS vary in different tumor cell lines, including inhibition of viability and proliferation, induction of expression of apoptosis-related genes, regulation of cytoskeleton structure and activation of multiple intrinsic and extrinsic apoptosis/autophagy pathways of tumor cells. Also, TCS exploits different antivirus approaches toward various viruses. Although the mechanism of action of TCS has not yet been solved, there are ongoing efforts to further modify TCS to promote its pharmacological properties and explore the potential medicinal applications of TCS in cancer immunotherapy. TCS may also have potential therapeutic value for transplantation rejection and other inflammatory diseases for its immunomodulatory effects. Further research on the mechanism and the pharmacological application of TCS will not only increase the translational values of this important protein, but the effort may also be extended to RIPs found in other plants, bacteria and mushrooms.

## Figures and Tables

**Table 1:** The anti-tumor activities of trichosanthin, including in vitro cell lines and in vivo animal models.

System	Tumor Type	Tested Cell Line	Tested Model
Female reproductive	Breast cancer	MCF-7, BT-474 and MDA-MB-231 [60]	Nude mice [60]
	Cervical cancer	HeLa [78] and Caski cells [79]	-
	Choriocarcinoma	JAR [59] and BeWo [80]	-
Immune	Lymphoma	SU-DHL-2 cells [61]	-
Digestive	Colon cancer	CT-26 [81]	-
	Hepatoma	HepA-H cells [81]	-
	Gastric cancer	MCG803 [82]	-
Blood	Leukemia	HL-60 [83] and K562 [84]	-
Respiratory	Lung cancer	A549 cells [85] and 3LL [86]	Nude mouse [85] Lewis rat murine models [86]
	Nasopharyngeal cancer	CNE1 and CNE2 [87]	-
Male reproductive	Prostate cancer	RM-1 [88]	-
Integumentary	Melanoma	B16 [89]	-
Nervous	Glioma	U87 and U251 [90]	-
